# Pasteurized *Akkermansia muciniphila* Timepie001 ameliorates DSS-induced ulcerative colitis in mice by alleviating intestinal injury and modulating gut microbiota

**DOI:** 10.3389/fmicb.2025.1542522

**Published:** 2025-02-19

**Authors:** Huan Han, Hui Xiong, Zengli Liu, Xunzhi Liu, Hailin Wang, Jiaxiang Kou, Dewei Yi, Ying Shi, Hao Wu, Jianjun Qiao

**Affiliations:** ^1^School of Chemical Engineering and Technology, Tianjin University, Tianjin, China; ^2^Zhejiang Institute of Tianjin University, Shaoxing, China; ^3^Shanghai Fuyang Biotechnology Co., Ltd., Shanghai, China; ^4^College of Food and Bioengineering, Fujian Polytechnic Normal University, Fuqing, China

**Keywords:** *Akkermansia muciniphila*, Timepie001, ulcerative colitis, gut microbiota, intestinal regulation

## Abstract

**Introduction:**

*Akkermansia muciniphila* (*A. muciniphila*), known as a next-generation probiotic, has been widely recognized for its beneficial effects in various metabolic diseases. While there is not much research whether live or pasteurized *A. muciniphila* has different effects on intestinal health.

**Methods:**

In the present study, a strain of *A. muciniphila* was isolated from healthy individuals, with the live and pasteurized *A. muciniphila* named Timepie001 and Timepie001+, respectively. They were administered to dextran sulfate sodium-induced ulcerative colitis mice to investigate their influences on the host intestinal health.

**Results and conclusion:**

The results showed that prophylactic supplementation with live and pasteurized *A. muciniphila* alleviates ulcerative colitis symptoms by retarding weight loss, preserving intestinal tissue structure, modulating inflammatory cytokines (TNF-α, IL-1β), and enhance the colonic mucosal barrier by upregulating the expression of tight junction protein Claudin-1. Interestingly, pasteurized *A. muciniphila* has a better effect compared with live *A. muciniphila*. Moreover, pasteurized *A. muciniphila* can regulates the gut microbiome to maintain intestinal homeostasis. This provides theoretical support for the widespread application of postbiotics in the food industry.

## 1 Introduction

Ulcerative colitis (UC) is a chronic inflammatory bowel disease (IBD) commonly known as “green cancer.” The pathogenesis of UC is complex and not fully understood, involving oxidative stress, intestinal mucosal damage, inflammation, and microbial dysregulation (Han et al., 2023; Seni et al., 2024). Lesions typically occur in the sigmoid colon and rectum but can extend to the descending colon, or even the entire colon (Li Y. et al., 2024). UC has become a global health problem seriously affecting the quality of life of patients (Zheng et al., 2023). The etiology of UC is multifactorial, arising from the interplay of genetic predisposition, environmental factors, unhealthy dietary habits, and autoimmune responses (Ni et al., 2017). Current treatments for UC include corticosteroids, immunosuppressants, and aminosalicylates, which despite their effectiveness, often come with significant costs and adverse side effects (Wangchuk et al., 2024). Therefore, there is a pressing need to further elucidate the underlying pathogenesis of UC and to develop safe and effective therapeutic strategies.

At present, although the pathogenesis of UC is not fully understood, an increasing number of studies indicate that the disrupted intestinal environment and gut microbiota are key factors affecting the occurrence and development of UC (Guo et al., 2021; Cheng et al., 2023; Zhang et al., 2025). Probiotics can modulate the balance of the gut microbiome, influence the metabolites produced by gut microorganisms, and play a key role in human intestinal health (Wang et al., 2024; Zhang et al., 2024). Multiple studies indicate probiotics can inhibit the proliferation of pathogenic bacteria, maintain mucosal barrier function, protect the host from pathogen-induced damage, promote the development of the intestinal immune system, and participate in immune regulation through the production of beneficial metabolites (Wu et al., 2022; Dai et al., 2024). This contributes to establishing a healthy gut microenvironment that can prevent and alleviate enteritis (Shen et al., 2018). Compared to healthy individuals, Earley et al. (2019) quantitatively analyzed the amount of *A. muciniphila* in mucus brushings from the colonic mucus, and found a significant reduction in all four areas of the colon, the caecum, transverse colon, left colon and rectum in patients with UC. Vigsnaes et al. (2012) and Wang et al. (2020) confirmed that *A. muciniphila* in stool samples was significantly reduced in patients with UC. The DSS-induced acute UC model in mice is a commonly used animal model for studying UC. Similarly, numerous studies have shown that *A. muciniphila* in animals with experimental colitis is negatively correlated with disease severity (Berry et al., 2012; Dunn et al., 2016; Zhang et al., 2016). These findings suggest that *A. muciniphila* may play a role in the pathogenesis and progression of UC and could be a potential target for therapeutic strategies.

*A. muciniphila* is a normal human intestinal bacterium and a strictly anaerobic gram-negative bacterium, oval-shaped organism, with 0.6–1.0 mm cell size. It is an obligate anaerobic, non-spore forming, and non-motile, belonging to the Verrucomicrobia phylum, was first isolated in 2004 by Derrien et al. (2004) from the feces of a healthy individual. *A. muciniphila* colonizes the human intestinal mucosal layer, where it degrades mucin and regulates the intestinal barrier and immune response. The abundance of *A. muciniphila* in the human intestinal tract represents 1–4% of the total fecal microbiota, and changes with age, diet, body weight and immune state throughout a life-time (Sharon et al., 2018). As the next generation of probiotics, *A. muciniphila* have been reported various beneficial properties, including protective effects against metabolic and diabetes, obesity, pulmonary fibrosis, heredity and immune conditions, colitis-associated colorectal cancer (Payahoo et al., 2019; Zhao et al., 2023; Faghfuri and Gholizadeh, 2024; Yu et al., 2024). Moreover, both animal experiments and human trials have demonstrated that exogenous supplementation with *A. muciniphila* or pasteurized *A. muciniphila* is effective, safe, and tolerated (Frank et al., 2007; Zhai et al., 2019). Depommier et al. (2019) conducted a randomized, double-blind, placebo-controlled pilot study in overweight/obese insulin-resistant volunteers. After 3 months of supplementation, they found that oral administration of pasteurized *A. muciniphila* improved insulin sensitivity by 30% compared to the placebo group, reducing insulin levels and total plasma cholesterol, while live bacteria did not exhibit these effects. The previous articles mainly focused on the field of metabolic disease, with limited research on UC. It is interesting that sometimes researchers’ conclusions are contradictory. Such as, Qu et al. (2021) found that *A. muciniphila* strain BAA-835 significantly ameliorated the symptoms in DSS-induced acute colitis; Bian et al. (2019) pointed that *A. muciniphila* Muc^T^ can protect the gut barrier function and reduce the levels of inflammatory cytokines to ameliorates dextran sulfate sodium (DSS)-induced UC in mice. However, Seregin et al. (2017) investigated fecal samples from two genetically colitis-prone mouse model, namely IL10^−/−^ and Winnie^−/−^ mice. Both models exhibited more abundant *A. muciniphila* than their wild-type littermates, and gavage of *A. muciniphila* to IL-10^−/−^ mice aggravated their colitis. Therefore, further research is needed to investigate the effect of *A. muciniphila* on colitis.

In this study, a strain of *A. muciniphila* was first screened and identified from healthy individuals and designated as Timepie001, while its pasteurized form was named Timepie001+. The optimal effects of live and pasteurized *A. muciniphila* intervention, as well as prophylactic supplementation, on intestinal barrier function in DSS-induced acute UC mice were then investigated. Additionally, possible mechanisms were explored through changes in inflammatory factors, expression of intercellular junction proteins and the gut microbiota.

## 2 Materials and methods

### 2.1 Preparation of Timepie001

A fecal sample from a healthy adult volunteer was freshly collected in a polyethylene bag and approximately 0.2 g fecal sample was dissolved in anaerobic PBS (pH7.2) containing 0.5 g/L of cysteine-HCL. The suspension was thoroughly mixed and serially diluted in 10-fold increments. Diluted suspensions were streaked onto brain heart infusion (BHI) agar plates supplemented with 2.5 g/L mucin. The plates were incubated at 37°C under anaerobic conditions generated by a gas mixture of 182 kPa N_2_/CO_2_/H_2_ (85:10:5, v/v) for 3 days. Single colonies were then selected and re-streaked onto fresh BHI plates containing 2.5 g/L mucin until pure cultures were obtained.

To determine the phylogenetic affiliation of the purified colonies, nucleotide sequence analysis of the cloned 16S rRNA gene was performed (Gómez-Gallego et al., 2016). The 16S rRNA gene sequence was compared to sequences from GenBank using the program BLASTN 2.0, available through the National Centre for Biotechnology Information (NCBI) website.^1^

*A. muciniphila* was incubated at 37°C for 48 h in BHI medium contained 2.5 g/L mucin. Following incubation, *A. muciniphila* and pasteurized *A. muciniphila* (70 degrees Celsius for 30 min) subsequently centrifuged at 8,000 × g for 15 min at 4°C. The cells were washed twice by PBS and centrifuged. The washed cells were resuspended in protective medium containing 7% (w/v) skim milk, 5% (w/v) trehalose and 1.5% (w/v) natrascorb as lyoprotectors. Immediately, the cells were prefrozen at −40°C for 12 h, and then frozen at −15°C, 5 Pa for 24 h using a vacuum freeze dryer (Turuvekere Sadguruprasad and Basavaraj, 2018).

### 2.2 Cell culture and anti-inflammatory activity

RAW 264.7 cells were cultured in RPMI 1640 media with 10% fetal bovine serum (FBS), 1% penicillin-streptomycin (Cytiva, SH40003.01) in an environment containing 5% CO_2_ at 37°C. Once cell adhesion reached 80–90%, the cells were passaged at 1:2 ratios.

For experiments, cells were seeded into a 96-well or 6-well plate until they reached an amount of 2 × 10^6^ cells/mL. After 24 h incubation, the cells were treated by different percentages (50, 25 and 12.5%) of live *A. muciniphila*. Cell viability was assessed using the Cell Counting Kit-8 (CCK-8) (TargetMOI, C0005). The cells were then treated with 2 μg/mL lipopolysaccharides (LPS) (Solarbio, L8880) and 25% of live and pasteurized *A. muciniphila*. After an additional 24 h of incubation, the production of nitric oxide (NO), interleukin-1β (IL-1β) and interleukin-6 (IL-6) were measured using the Griess assay (Beyotime, S0021S) and ELISA kits (Excell, EM004-96/EM001-96).

### 2.3 Ulcerative colitis model and treatments

The animal experimental design was drafted according to the requirements of the Guide for the Care and Use of Laboratory Animals: Eighth Edition (Astronomy and Astrophysics, 1996). Healthy male age-matched C57BL/6J mice (6–8 weeks of age) were purchased from Zhejiang Academy of Medical Sciences (Hangzhou, China). DSS (mol wt, 36,000–50,000 Da, CAS No. 9011-18-1) was purchased from MP Biomedicals (Goddard, United States). All animals were housed under specific pathogen-free conditions in IVC cages with 12 h light/dark cycles, enriched water and food feeding. Following 1 week of acclimatization, the mice were randomly assigned to four groups (*n* = 6 for each group).

Negative control group (NC): received deionized water for 7 days.
DSS group (DSS): 2.5% DSS was given as the sole source of drinking fluid and administered PBS for 7 days.
Live *A. muciniphila* (Timepie001) group: 2.5% DSS was given as the sole source of drinking fluid and administered live *A. muciniphila* (1 × 10^9^ CFU/mouse) by oral gavage for 7 days.
Pasteurized of *A. muciniphila* (Timepie001+) group: 2.5% DSS was given as the sole source of drinking fluid, and pasteurized *A. muciniphila* (same weight as live *A. muciniphila*) was administered by oral gavage for 7 days.

During the duration of the experiment, the body weight, fecal status, and degree of fecal blood were measured daily for disease activity index (DAI) assessment. At the end of the experiment, spleens were excised, rinsed with 0.9% NaCl, and weighed. Colons were collected, rinsed with ice-cold 0.9% NaCl, placed on filter paper, and their lengths were measured using a ruler. Each colon was then divided into four large sections, which were snapped frozen at −80°C. The above process was inspected and approved by the Animal Care & Welfare Committee of Zhejiang University of Technology, China (No. ZH20240531066).

### 2.4 Disease activity index

The disease activity index (DAI) was assessed based on body weight loss, diarrhea, and hematochezia to evaluate the severity of colitis. Body weight loss was scored as follows: 0 (≤1%), 1 (1–5%), 2 (5–10%), 3 (10–15%), and 4 (≥15%). Diarrhea and rectal bleeding were scored daily, with rectal bleeding rated as: 0 (no bleeding), 2 (slight bleeding), and 4 (extensive bleeding); diarrhea was scored as: 0 (well-formed stools), 2 (soft and pasty stools), and 4 (watery stools). The average DAI value was calculated from these scores.

### 2.5 Assessment of colon inflammation

Colonic tissues were fixed in 4% paraformaldehyde solution at room temperature and then embedded in paraffin. The fixed colon was cut into 5 μm sections for hematoxylin and eosin (H&E) staining. Pathological analysis of all sections was performed using light microscopy. The specific evaluation criteria are as follows: depth of inflammation and at a range from 0 to 4 as to the amount of crypt damage or regeneration. (0: intact recess without inflammation; 1: 1/3 recess damaged; 2: 2/3 recess damage and mild inflammation; 3: large recess damage, intact mucosa, and moderate inflammation; 4: mucosal epithelium loss, erosion, severe inflammation).

### 2.6 RNA extraction and RT-qPCR

RNA extraction from colonic tissue was modified from previously established methods (Cheng et al., 2021). Total RNA was extracted from the colon tissue samples with RNAprep Pure Tissue Kit (TIANGEN, DP431). The RNA purity was assessed with a microspectrophotometer (Mettler Toledo UV5) and 260/280 nm of all the samples were between 1.8 and 2.1. Following the kit’s instructions, RNA was reverse-transcribed into cDNA using the SPARKscript II RT Plus Kit (with gDNA Eraser) (SparkJade, AG0304-B). RT-qPCR amplification was performed using 2 × SYBR Green qPCR Mix (with ROX) (SparkJade, AG0104-B). The primer sequences for GAPDH, IL-1β, tumor necrosis factor-α (TNF-α) and zonula occludens-1 (ZO-1) were detailed in Table 1, with GAPDH serving as an internal reference gene. Relative mRNA expression levels were calculated using the $2^{-\mathrm{\Delta \Delta CT}}$ method.

**Table 1:** Primer sequence list of the tested genes.

Gene	Sense (5′–3′)	Anti-sense (5′–3′)
*GAPDH*	TGGCCTTCCGTGTTCCTAC	GAGTTGCTGTTGAAGTCGCA
*IL-1β*	TCGCAGCAGCACATCAACAAGAG	TGCTCATGTCCTCATCCTGGAAGG
*TNF-α*	CAGGCGGTGCCTATGTCTC	CGATCACCCCGAAGTTCAGTAG
*ZO-1*	GCGAACAGAAGGAGCGAGAAGAG	GCTTTGCGGGCTGACTGGAG

### 2.7 Western blot analysis

Western blot analysis was performed according to modified protocols from previous studies (Lee et al., 2022). Total proteins were extracted from the colonic tissue using RIPA lysis buffer (containing protease inhibitors) (Macklin, Y10001173) and sonicated at 4°C. Protein concentrations in tissue lysates were quantitated using a BCA protein assay kit (Solarbio, PC0020). After centrifuging the lysed sample, protein concentrations were adjusted, mixed with a 5× loading buffer, and heated in a metal bath for 10 min. Proteins (10 μL) were separated using 10% SDS-PAGE and transferred to polyvinylidene fluoride (PVDF) membranes (Millipore, Billerica, MA, United States). After completion, the membranes were blocked for 1 h with 5% BSA in 1% TBST and probed with specific primary antibodies [GADPH (Affinity, AF7021), Claudin-1 (Zenbio, 343202)] at 4°C overnight. The PVDF membrane was then washed five times with 1% TBST (TBS with Tween-20) and incubated with secondary antibodies (ZSGB-BIO, ZB-2301) in 5% bovine serum albumin (BSA) at room temperature for 1 h. After the PVDF membrane was washed five times, immunoblot images were captured using a fluorescence imaging system (Tanon-5200Multi), and the band expression levels were quantified using Image J. GAPDH was used as a reference gene.

### 2.8 Taxonomic analyses of the gut microbiota

Fresh fecal samples were collected in sterile sampling tubes, with approximately 200 mg from the distal segment taken using a sampler and placed into a tube containing nucleic acid preservative solution, then immediately stored at −80°C until DNA extraction. Genomic DNA was extracted from all fecal samples and amplified using polymerase chain reaction (PCR) with primers specific to the 16S V3–V4 region. Libraries were constructed using the Illumina Inc. library preparation kit (Model: TruSeq DNA PCR-Free Library Preparation Kit). After quantitative assessment and qualification, sequencing was performed on the NovaSeq 6000. Sequencing reads were assembled, and sequence quality was controlled and filtered. The sequences were clustered into operational taxonomic units (OTUs) with 97% similarity and annotated using the Silva 138 database. Species abundances across different categorization levels were detected together with the composition of the gut microbiota in each sample at each level of classification (phylum, class, order, family, and genus). The number of OTUs in each group was statistically analyzed to compare the changes in the richness of the mouse intestinal microbial community. Alpha diversity was assessed using the Shannon, Simpson, Chao1, and ACE index. The composition of the microbial community was illustrated in a bar chart at the genus level and differences were analyzed.

### 2.9 Statistics analysis

The data are presented as Mean ± SEM with at least triplicates and *n* = 6 for each group. All analyses were performed using Graphpad Prism version 5.0 (Graphpad Software, San Diego, CA, United States). One-way analysis of variance (ANOVA) was utilized to compare multiple groups. When significant differences were identified, Tukey’s *post hoc* test was applied to determine specific differences between pairs of means, with appropriate adjustments for multiple testing. In all analyses, the confidence interval was set at 95%, and *p*-values less than 0.05 were considered statistically significant.

## 3 Results

### 3.1 Isolation and identification of *Akkermansia muciniphila*

**Figure 1:**
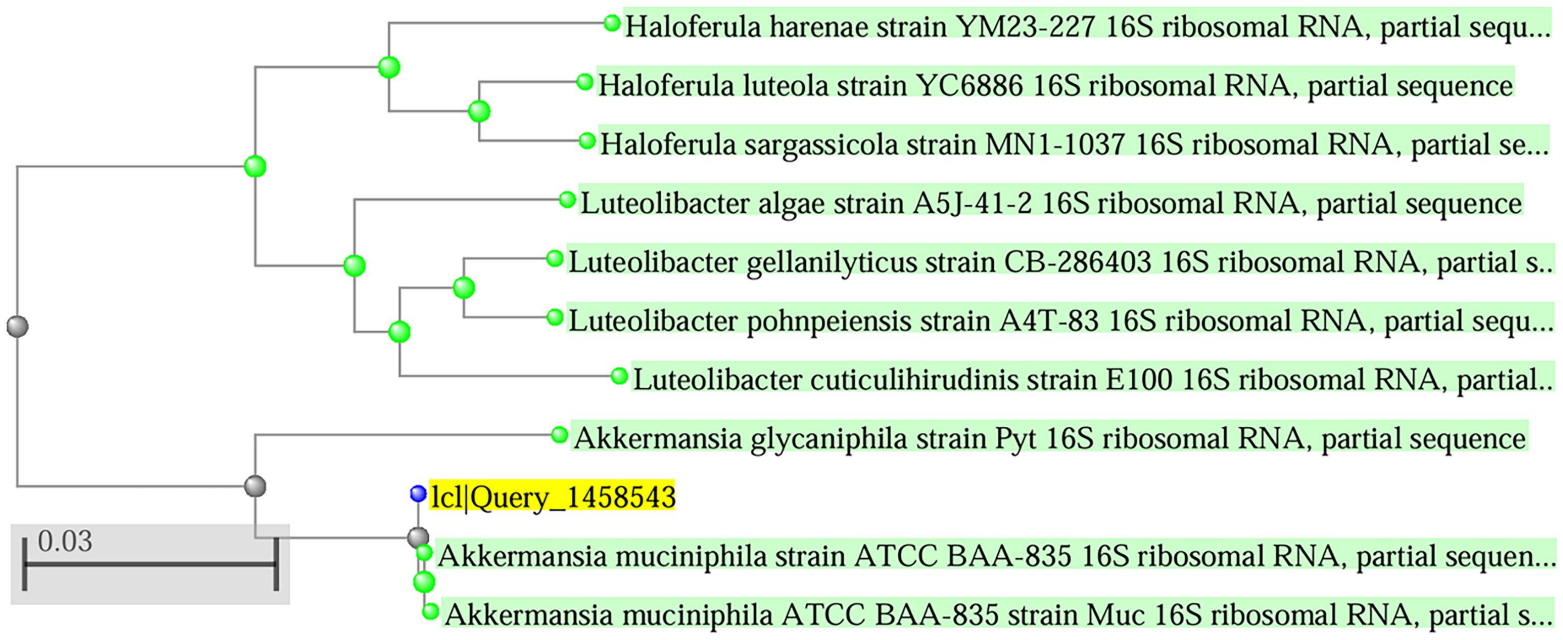
Phylogenetic tree showing the position of the determined 16S rDNA gene sequence of the strain purified among those of selected type strains. The tree was generated by the neighbor-joining method.

The strain *A. muciniphila* Timepie001 was isolated from healthy human subjects and identified through 16S rDNA analysis, which revealed a 99.93% similarity with the 16S sequence of *A. muciniphila* ATCC BAA-835, confirming its classification as *A. muciniphila* (Ouwerkerk et al., 2016). To further establish the evolutionary relationship between the two strains, whole-genome sequencing of Timepie001 was conducted, followed by average nucleotide identity (ANI) analysis comparing it to *A. muciniphila* ATCC BAA-835 (Figure 1). The ANI value obtained for Timepie001 was 97.24%, meeting the ANI threshold for species classification (greater than 95%). Subsequent experiments utilized pasteurized freeze-dried powder of the strain.

### 3.2 Anti-inflammatory activity *in vitro*

**Figure 2:**
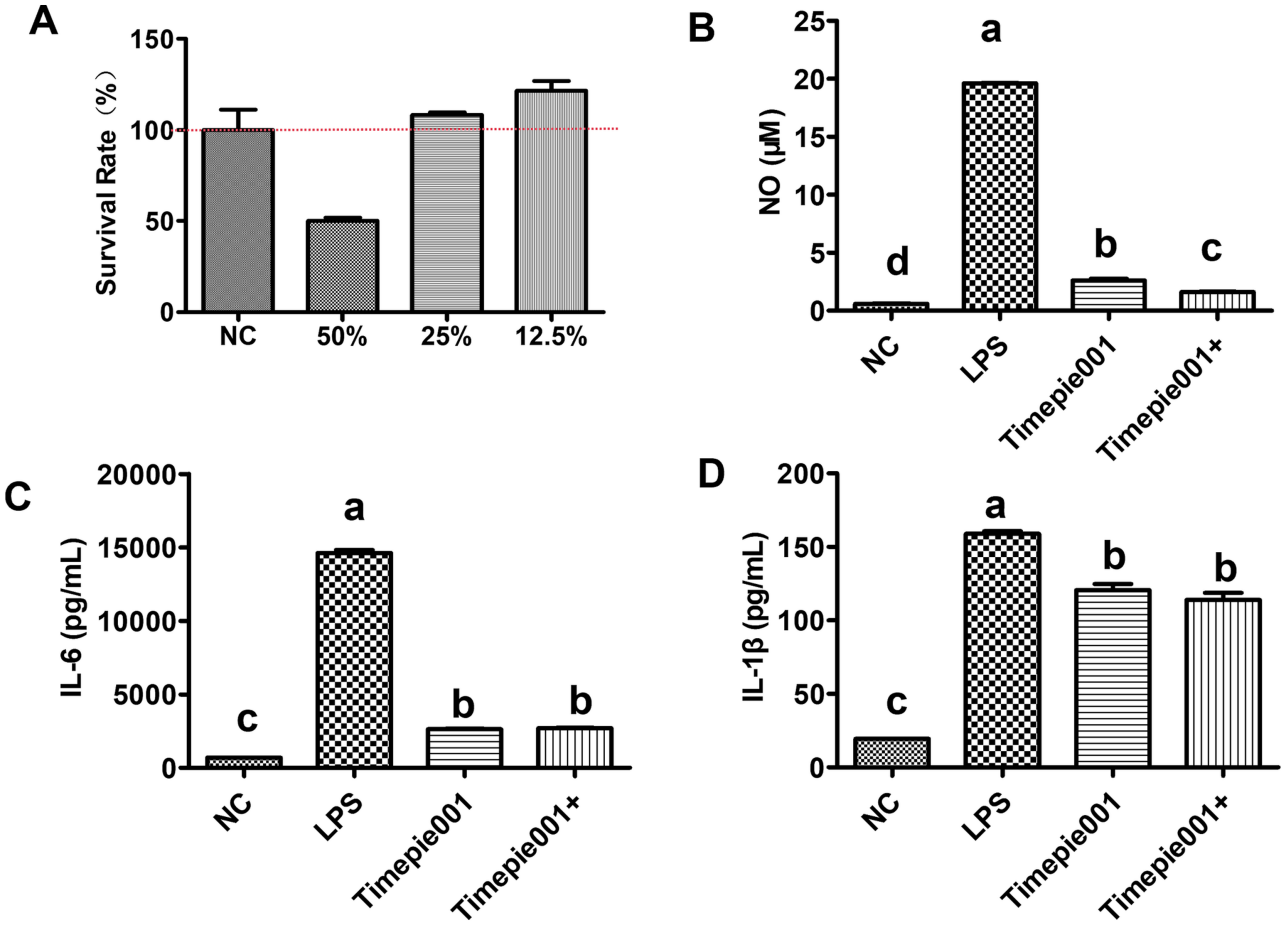
The effect of Timepie001 and Timepie001+ on the survival rate and secretion of pro-inflammatory cytokines in RAW264.7 cells. **(A)** Effects of different concentrations of Timepie001 on survival rate of RAW264.7 cells. **(B–D)** Concentrations of the NO, IL-6, and IL-1β. The results were expressed as the mean values ± SEM (*n* = 3). One-way ANOVA was used for statistical analysis, followed by Tukey’s *post hoc* test. Different letters indicate significant differences (*p* ≤ 0.05).

Macrophages are important participants in the pathogenesis of UC due to their production of many pivotal cytokines. In this study, an LPS-induced RAW 264.7 cell model was established *in vitro*. Cell viability was assessed using the CCK-8 assay and ELISA was utilized to measure the protein levels of cytokines in the supernatant of different experimental groups. The survival rates of RAW264.7 cells treated with varying concentrations of Timepie001 for 24 h are shown in Figure 2. It is evident that Timepie001 at concentrations ranging from 12.5 to 25% exhibited no cytotoxicity, with high cell viability observed. For subsequent experiments, a concentration of 25% was selected for cellular treatment, corresponding to a Timepie001 concentration of 1 × 10^8^ CFU/mL. Figure 2 displays the effects of Timepie001 and Timepie001+ on the secretion of NO, IL-6 and IL-1β from LPS-induced RAW264.7 inflammatory cells. As illustrated in Figures 2B–D, LPS (2 μg/mL) significantly increased the secretion of NO, IL-6 and IL-1β (*p* < 0.01). Following intervention with Timepie001 and Timepie001+, compared with model group, the secretion levels of these three inflammatory factors were significantly reduced by 86.66, 81.99, 24.20 and 91.80%, 81.61 and 28.40%, respectively (*p* < 0.05). These results indicate that Timepie001 and Timepie001+ markedly inhibits the inflammatory response in LPS-stimulated RAW 264.7 cells, Timepie001+ has better anti-inflammatory effects.

### 3.3 Body weight and DAI scores

**Figure 3:**
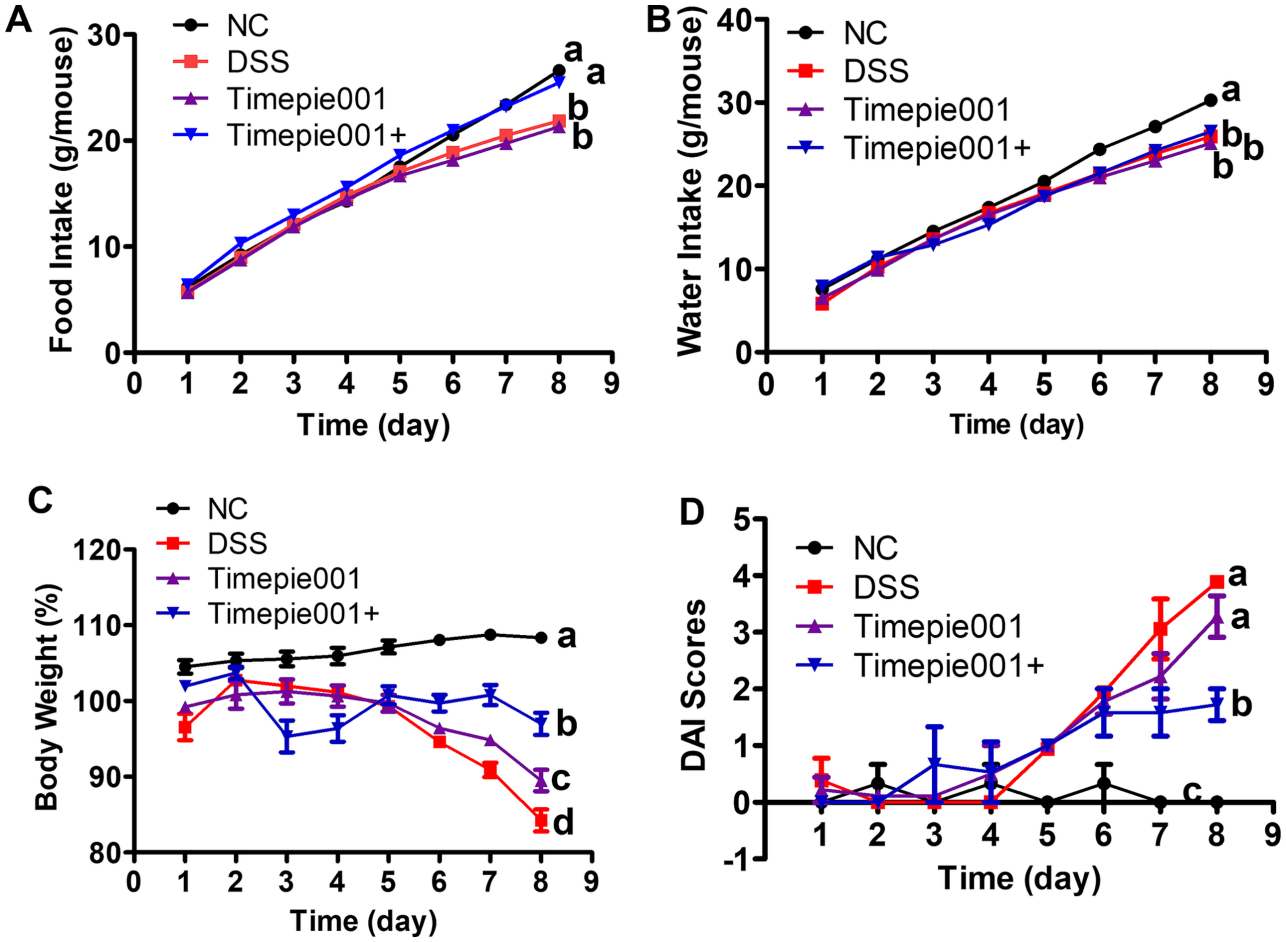
Effect of Timepie001 and Timepie001+ on the average. **(A)** Total food. **(B)** Total water intake of each mouse. **(C)** Daily body weight variation and changes in **(D)** DAI scores with DSS-induced colitis in 8 days. The mean ± SEM are shown (*n* = 6). ^a–d^Values followed by different letters indicate significant differences (*p* ≤ 0.05) between different group using one-way ANOVA followed by Tukey’s test for *post hoc* analysis.

Food and water intake generally serves as indicator of an organism’s physiological status. As shown in Figures 3A,B, there was no significant difference in water and food intake among the four groups in 4 days. However, significant differences in food and water intake were observed among the group on the 8th day. In terms of total food intake, there was no significant difference between the Timepie001+ group and NC group (*p* > 0.05), and no significant difference between the Timepie001 group and DSS group on the 8th day. Regarding total water intake, significant differences were found between the other three groups and the NC group (*p* < 0.05), but no significant difference was ovserved among the three groups (*p* > 0.05). These results indicate that some pathological changes occurred in the three groups of mice after 5 days, the condition of mice in Timepie001+ group was slightly better than that in the Timepie001 group.

The current findings successfully established a DSS-induced mouse model of UC, characterized by typical symptoms including weight loss, diarrhea, hematochezia, and an increase in the DAI. During the experiment, the body weight of the NC group mice continued to increase over time. In contrast, both the Timepie001, Timepie001+ and DSS groups showed a significant reduction in body weight compared to the NC group mice (*p* < 0.05). By day 8, the body weight of DSS-induced mice had decreased by 15.77% from their initial weight. However, compared to the DSS group, the weight loss in the Timepie001 and Timepie001+ group were significantly reduced (Figure 3C), suggesting that Timepie001 and Timepie001+ effectively alleviates weight loss in DSS-induced mice. The DAI score was used to evaluate the impact of Timepie001 on colitis in mice. As illustrated in Figure 3D, the DAI index of DSS group mice increased due to DSS administration compared to the NC group, whereas the DAI index of mice receiving oral Timepie001+ was significantly reduced. The Timepie001 group did not show a similar effect.

### 3.4 Colon length and spleen distension size

**Figure 4:**
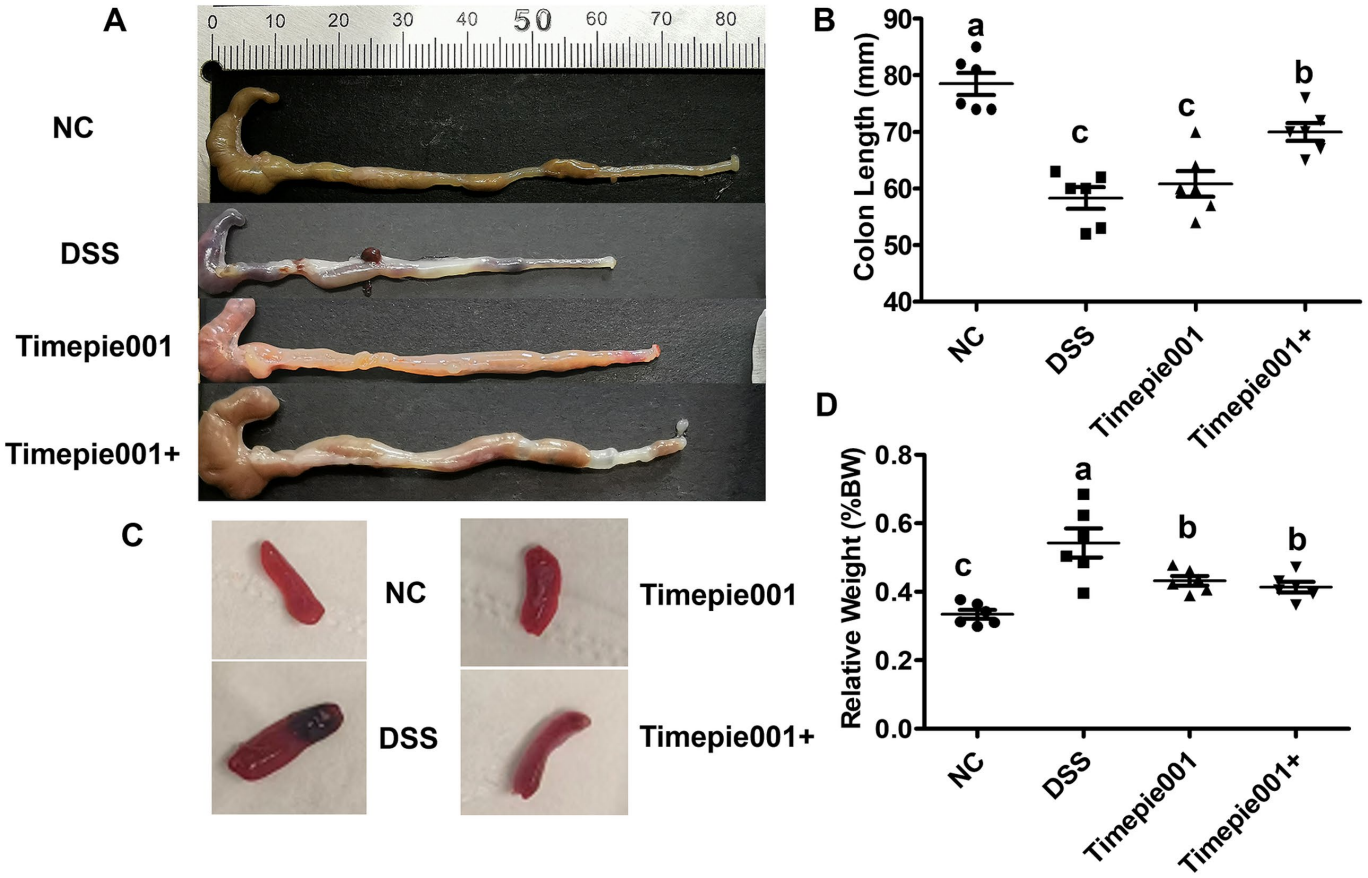
Effect of Timepie001 and Timepie001+ on the symptoms changes of colitis mice. **(A)** Macroscopic pictures of colons. **(B)** Colon length. **(C)** The morphology of the spleen. **(D)** Relative spleen weight (%BW). The values are presented as means of six mice ± SEM. ^a–c^Values followed by different letters indicate significant differences (*p* ≤ 0.05) between different group using one-way ANOVA followed by Tukey’s test for *post hoc* analysis.

As shown in Figure 4A, the colon tissue of the NC group exhibited normal color and thickness, with no signs of edema or congestion, and no adhesions to surrounding tissues. In contrast, mice treated with DSS displayed clear symptoms of UC, including congestion, edema, shortened colon length, as well as ulcers, localized necrosis, and melena. Notably, Timepie001+ was found to effectively alleviate colonic damage in DSS-induced colitis models. The shortening of the colon is typically associated with inflammation, and it is important to highlight that both Timepie001 and Timepie001+ significantly reduced colon shortening in colitis mice compared to the NC group, with the improvement effect of Timepie001+ being better (Figure 4B). The spleen, as the largest lymphatic organ, contains a high number of lymphocytes and macrophages involved in both humoral and cellular immune responses. Inflammatory conditions lead to splenomegaly. The normal spleen appears smooth and deep red; however, the spleens of DSS-treated mice became enlarged and darker. Following Timepie001+ treatment, the size and color of the spleens were comparable to those of the NC group (Figure 4C). The relative spleen weight of DSS-treated mice significantly increased (*p* < 0.05, as shown in Figure 4D). Timepie001 and Timepie001+ markedly countered the enlargement of the spleen, indicating its inhibitory effect on the peripheral inflammation associated with colitis. There was no significant difference in the relative weight of the spleen between Timepie001 and Timepie001+.

### 3.5 Histopathological alterations

**Figure 5:**
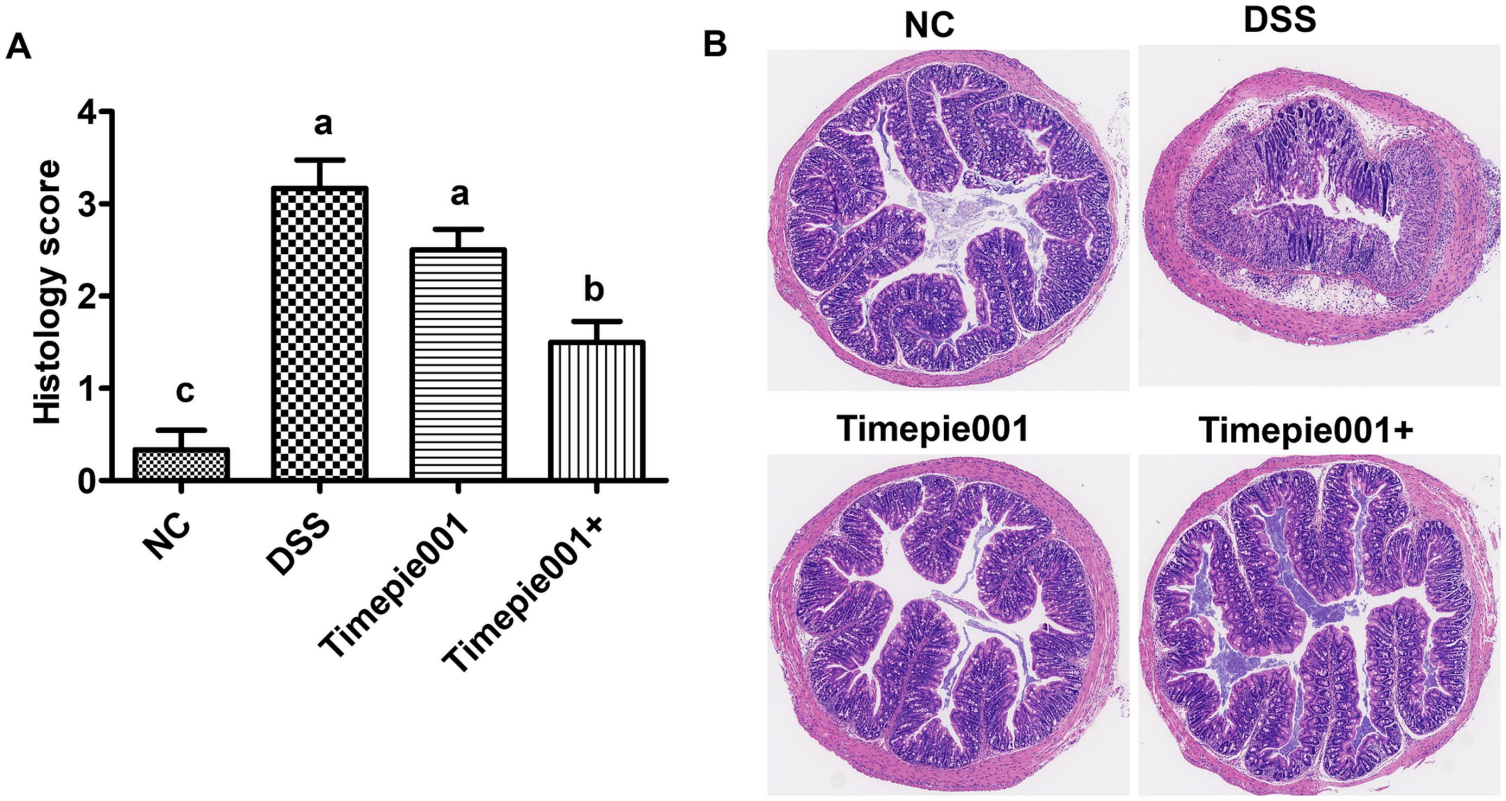
Effects of Timepie001 and Timepie001+ on histopathology changes of colon tissues in colitis mice. **(A)** The colonic histopathological score. **(B)** H&E staining (100×). The values are presented as means of 6 mice ± SEM. ^a–c^Values followed by different letters indicate significant differences (*p* ≤ 0.05) between different group using one-way ANOVA followed by Tukey’s test for *post hoc* analysis.

The degree of colonic damage, including the status of the intestinal wall, crypt integrity, colonic tissue ulceration, and neutrophil infiltration, can be assessed using H&E staining. The effect of Timepie001 and Timepie001+ on colonic histology in colitis-affected mice is illustrated in Figure 5B. The colon mucosa of the NC mice was intact, with well-distributed glands and no ulceration or significant inflammatory cell infiltration. In the DSS group, the structure of the intestinal mucosa was disrupted, resulting in irregular shapes, sloughing, ulceration, obvious inflammatory cell infiltration and a significant increase in the histological score. After treatment with Timepie001 and Timepie001+, the colonic tissue injury of mice was significantly improved. In the Timepie001+ group, the area of mucosal ulcers in the colonic tissue was reduced, and the intestinal wall showed signs of restoration (Figure 5B). Additionally, histological examination revealed that mice in the Timepie001+ group had reduced immune cell infiltration and less disruption of the crypts, along with lower histological scores compared to the DSS group (Figure 5A). However, there was no significant difference in histological scores between the DSS group and the Timepie001 group (*p* > 0.05). These results indicate that treatment with Timepie001+ can ameliorate clinical symptoms, improve histological scores, and reduce inflammatory cell infiltration in a murine model of colitis.

### 3.6 Expression of related genes in colonic tissues

**Figure 6:**
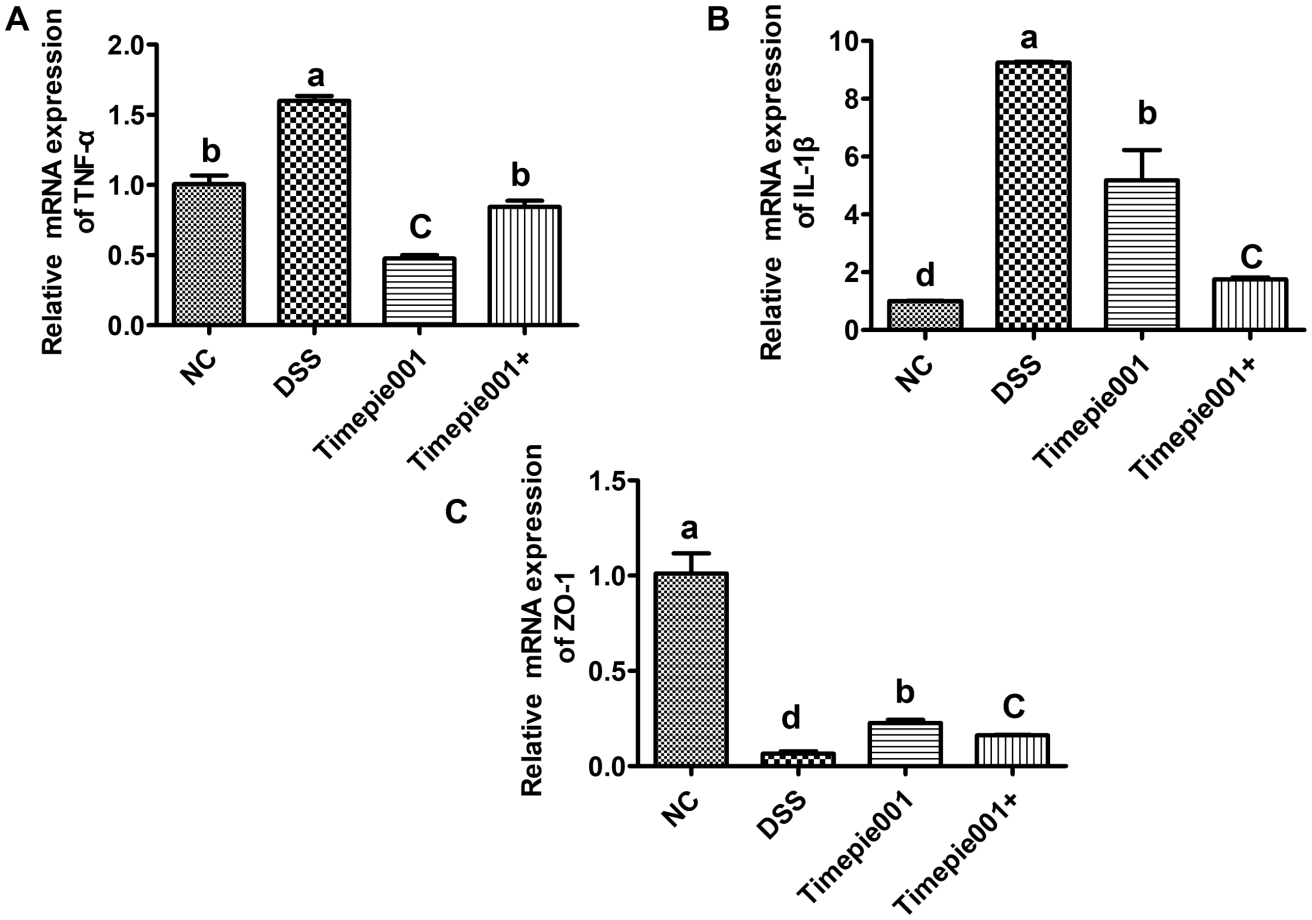
Effect of Timepie001 and Timepie001+ on expression of (TNF-α **(A)**, IL-1β **(B)**, and ZO-1 **(C)**) in colonic tissues of mice with UC induced by DSS. Dates are expressed as mean ± SEM. ^a–d^Values followed by different letters indicate significant differences (*p* ≤ 0.05) between different group using one-way ANOVA followed by Tukey’s test for *post hoc* analysis.

Quantitative RT-qPCR results for IL-1β, TNF-α and ZO-1 mRNA expression are presented in Figure 6. Compared to the NC group, the mRNA expression of IL-1β, TNF-α were significantly increased and ZO-1 was decreased in the colon tissues of DSS-induced UC mice (*p* < 0.01). Following intervention with Timepie001 and Timepie001+, the expression of IL-1β and TNF-α was significantly reduced compared to the DSS group, with relative decreases of 44.02, 70.27 and 81.05%, 47.16%, respectively, while ZO-1 increased by 246 and 147% (*p* < 0.05). Inflammation is a major contributor to colon damage in UC. When colitis occurs, the intestinal barrier is disrupted, pathogens infiltrate the intestinal mucosa, causing a flood of pro-inflammatory cytokines to be released, which exacerbates the inflammatory response (Neurath, 2014). TNF-α and IL-1β are key pro-inflammatory cytokines that are released at elevated levels in patients with UC, intensifying the inflammatory response. These findings suggest that Timepie001+ can improve DSS-induced colitis by modulating the levels of inflammatory cytokines. Science UC is generally accompanied by an increase in intestinal permeability, and the increase in ZO-1 gene expression also indicated that Timepie001 and Timepie001+ can improve the intestinal permeability of the colon. With the improvement effect of Timepie001+ being more significant.

### 3.7 Expression of epithelial intercellular junction proteins

**Figure 7:**
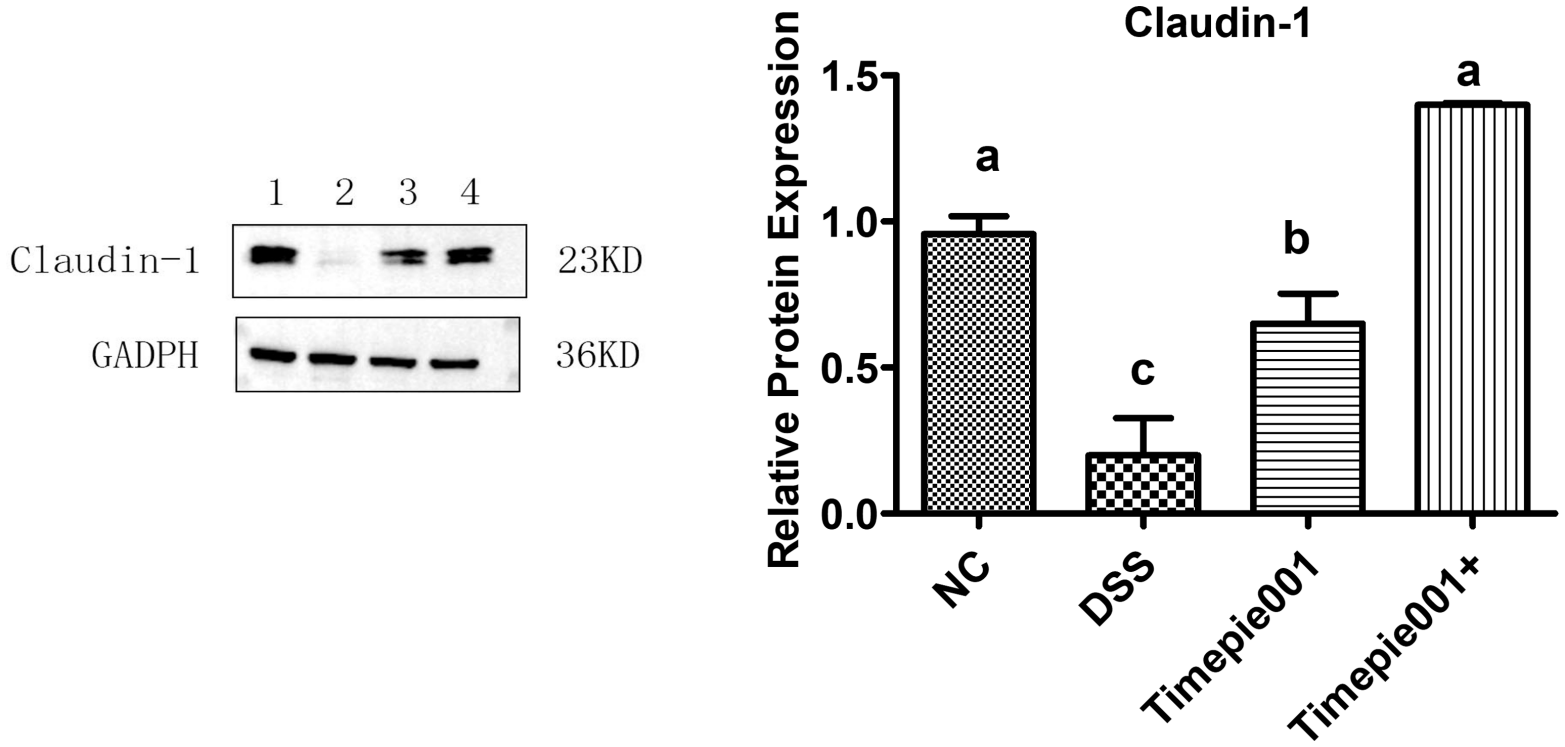
The relative protein levels of Claudin-1 in the mice colon. Dates are expressed as mean ± SEM. ^a–c^Values followed by different letters indicate significant differences (*p* ≤ 0.05) between different group using one-way ANOVA followed by Tukey’s test for *post hoc* analysis.

Claudin-1 protein is a crucial component of the colonic epithelial barrier. Pathogens and viruses can exploit tight junctions to infiltrate and infect cells, leading to the production of inflammatory cytokines. The disruption of the colonic epithelial barrier is a significant factor contributing to colitis. This study investigates the impact of Timepie001 and Timepie001+ on the expression of tight junction proteins in the intestines of mice with UC. As shown in Figure 7, there is a down-regulation of Claudin-1 expression in the colon of DSS-induced UC mice. However, although Timepie001 and Timepie001+ up-regulates Claudin-1 expression in UC mice (*p* < 0.05), there was no significant difference in protein expression levels between the Timepie001+ groups and the NC group. Overall, these finding suggest that Timepie001+ may mediate its protective effects against UC mice by enhancing intestinal barrier function.

From all the above results, it can be concluded that Timepie001+ group has a better improvement effect on DSS-induced UC. Therefore, in the following section on gut microbiota analysis, only the differences between the Timepie001+ group, NC group and DSS group were analyzed.

### 3.8 Gut microbiological analysis

**Figure 8:**
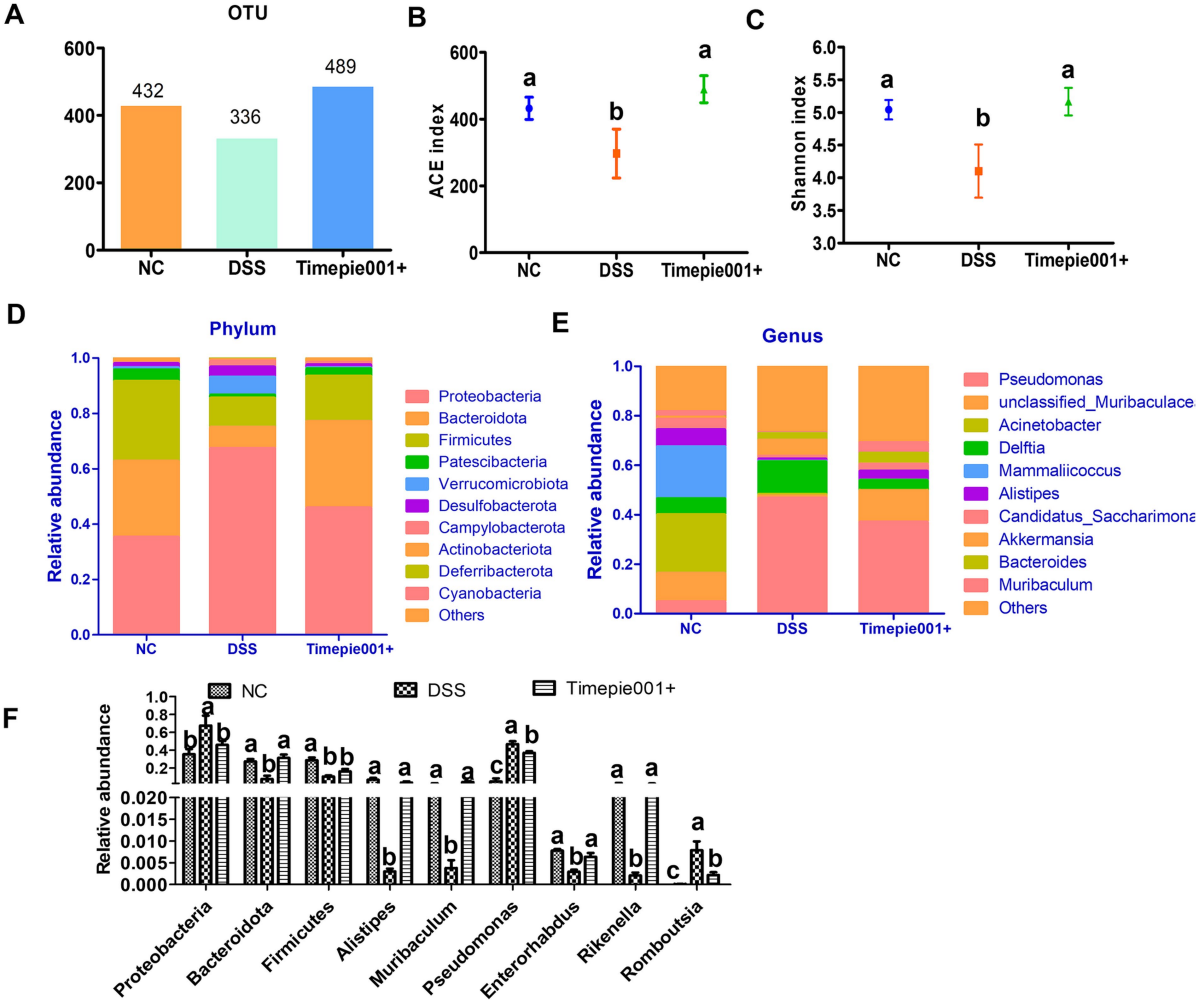
The effect of Timepie001+ on gut microbiota in colitis mice. **(A)** OTUs of gut microbiota. **(B,C)** The ACE index and Shannon index among three groups. **(D,E)** Relative abundance of taxa at the phylum, genus levels. **(F)** Relative abundance of taxa in the three groups. The values are presented as means of six mice ± SEM. ^a–c^Values followed by different letters indicate significant differences (*p* ≤ 0.05) between different group using one-way ANOVA followed by Tukey’s test for *post hoc* analysis.

The intestinal microbiota of mice with colitis often changes, and in this study, 16S rRNA gene sequencing was used to assess the composition of the intestinal microbiota. The number of OTUs in the sample detection results can reflect changes in the richness of the mouse intestinal microbial community. The NC group, DSS group and Timepie001+ group had 432, 336, and 489 OTUs, respectively (Figures 8A). The Timepie001+ group was closer to the NC group than the DSS group at the OTU level, indicating that the intestinal microbiota of the mice tended to develop at a normal level after intervention with pasteurized *A. muciniphila*, and the diversity of the intestinal microbiota of the mice was restored.

Alpha diversity was compared among the three groups using ACE and Shannon index (Figures 8B,C), where the ACE index reflects species richness, and the Shannon index reflects both richness and evenness. The results indicated significant differences in alpha diversity indices among the groups, with the ACE and Shannon values in the DSS group being markedly lower than those in the NC and Timepie001+ groups. The alpha diversity indices in the Timepie001+ group were comparable to the those of NC group, with both the ACE and Shannon indices slightly exceeding those of the NC group.

Metagenomic techniques were employed to determine the composition of the gut microbiota in each group. The relative abundance of major gut microbiota in each group is represented by bar charts at the phylum and genus level (Figures 8D,E). Notable changes were observed in the composition of the gut microbiota among UC mice. At the phylum level, the abundance of Proteobacteria, Verrucomicrobiota, Desulfobacterota, Campylobacterota, and Deferribacterota increased in DSS-induced mice compared to the NC group, while Bacteroidota, Firmicutes, Patescibacteria, and Actinobacteriota decreased. The relative abundances of Bacteroidota in the NC, DSS, and Timepie001+ groups were 27.42, 7.61, and 31.29%, respectively, and for Firmicutes, they were 28.80, 10.51, and 16.28% (Figures 8D,F). Following Timepie001+ intervention, the abundance of harmful bacteria decreased while that of beneficial bacteria increased. At the genus level, DSS induction led to a decrease in the abundances of Muribaculaceae, Acinetobacer, Mammaliicoccus, Alistipes, Candidatus_Saccharimonas Enterorhabdus, Halomonas, Limosilactobacillus, Rikenella and Muribaculum, while Pseudomonas, Delftia, Akkermansia and Bacteroides increased. Timepie001+ intervention alleviated these changes (Figures 8E,F).

## 4 Discussion

Probiotics are active microorganisms that are beneficial to human health. They mainly exist in the human intestine and play many important roles and effects, including maintaining intestinal health, promoting digestion and absorption, enhancing immunity, improving constipation and diarrhea, and reducing cholesterol (Daglia et al., 2024; Wang D. et al., 2025; Yao et al., 2025). Postbiotics has been a rapidly growing topic in recent years (Wegh et al., 2019). Experts in the field of probiotics and prebiotics suggested a definition for the term “postbiotics” which was “preparation of inanimate microorganisms and/or their components that confers a health benefit on the host” (Eslami et al., 2024). Most previous studies have focus on the beneficial effects of probiotics and prebiotics on UC. However, postbiotics appear to be safer than probiotics. Since postbiotics consist of nonviable microbial cells, Interference inhibition by interferon, infections, or inflammatory responses known side effects of probiotics is lower with postbiotics.

*A. muciniphila* is known as the second-generation probiotic, It can utilizes glycoprotein mucin as its direct source of carbon and nitrogen to colonize the human intestinal mucosal layer (Zhou and Zhang, 2019), which means it has a unique survival advantage of being able to survive in nutrient deficiencies and harsh conditions (Gómez-Gallego et al., 2016). *A. muciniphila* have the characteristic of mucosal degradation and play important role in regulating the intestinal barrier and immune response, thus contributing significantly to human health and diseases. The observation of decreased levels of *A. muciniphila* in UC patients suggests that these bacterial may contribute to the pathogenesis and procession of UC, and could be potential target and promising strategy for UC therapy (Earley et al., 2019). However, its anaerobic biological characteristics limit its widespread application in dietary supplements. Research have shown that after 30 min of pasteurization at 70°C, *A. muciniphila* retains most of its beneficial effects. Pasteurized *A. muciniphila* was found to increase systemic energy expenditure and fecal energy excretion in diet-induced obese mice, as well as enhance intestinal epithelial renewal (Depommier et al., 2020). However, there are few articles on the effectiveness of using pasteurized *A. muciniphila* for UC.

DSS-induces UC symptoms characterized by weight loss, diarrhea, hematochezia, shortened colon length, colonic swelling, splenomegaly, and even death (Jang et al., 2019; Ma et al., 2020; Shi et al., 2024). Colon length, DAI scores and histological analysis showed that Timepie001+ significantly improved DSS-induced inflammatory infiltration, crypt damage, and mucosal injury. While Timepie001 did not show significant improvement. Based on our findings, we propose several potential mechanisms, including modulation of the inflammatory response, enhancement of the intestinal mucosal barrier, and reconfiguration of the gut microbiota.

Inflammation is a key factor in the onset of UC. The production of TNF-α and IL-1β can further amplify the inflammatory response, exacerbating UC (Hu et al., 2024; Li Y. et al., 2024). TNF-α is primarily produced by monocytes and macrophages and acts as a pro-inflammatory cytokine involved in systemic inflammation, stimulating acute inflammatory responses (El-Boghdady et al., 2023). It conveys signals to the cell nucleus via specific receptors on the cell membrane, promoting various complex biological activities such as cell proliferation, differentiation, immune regulation, and inflammation mediation. TNF-α promotes the secretion of endothelial cell adhesion factors, increases the permeability of epithelial tight junctions, and inhibits epithelial cell growth, leading to damage to the mucosa and extracellular matrix. Numerous studies have revealed the anti-inflammatory effect of *A. muciniphila* (Miao et al., 2024; Xiao et al., 2024). For instance, oral administration of *A. muciniphila* strain BAA-835 significantly ameliorated the symptoms in DSS-induced acute colitis, as evidenced by decreased body weight loss, colon length shortening, and colon histological inflammatory score. Zhai et al. (2019) studied and compared the characteristics of *A. muciniphila* ATCC BAA-835 and *A. muciniphila* strain 139, and reported interesting results. Both strains showed anti-inflammatory effects including inhibiting IL-8 production induced by TNF-α co-cultured HT-29 cells *in vitro* anti-inflammatory experiments. However, these two strains exhibited different functions in the DSS-induced UC model. Although these two strains alleviated colitis symptoms by down-regulating pro-inflammatory cytokines, only the DSS + ATCC group observed significant reductions in spleen weight and inflammation index compared to the DSS group. In our study, it can be observed that Timepie001+ can significantly reduce the production of NO, IL-1β, and IL-6 induced by LPS after interacting with macrophages RAW 264.7 cells, demonstrating good anti-inflammatory effects. We also observed a decrease in the expression of inflammatory cytokines (TNF-α and IL-1β) in colon tissue. These results suggest that the protective effect of Timepie001+ on UC may be achieved through anti-inflammatory effects.

UC with damage to the intestinal mucosal barrier being a critical pathological mechanism (Wang J. et al., 2025; Wang Y. et al., 2025). The intestinal mucosal barrier serves as the body’s first line of defense against harmful substances and pathogens, comprising chemical, biological, immune, and mechanical barriers, with the mechanical barrier playing a predominant role (Li J. et al., 2024). Previous reports indicate that the colons of UC patients or model mice are thinner compared to controls, and intestinal permeability is increased in colitis models. Madsen et al. (2010) explored the relationship between increased intestinal permeability and intestinal inflammation in IL-10 deficient mice, noting that at 2 weeks of age, these mice showed no signs of intestinal damage but exhibited increased intestinal permeability alongside elevated concentrations of TNF-α and IFN-*γ*. This suggests that the increase in intestinal permeability precedes the inflammatory response. Tight junction proteins are essential components of the intestinal mucosal mechanical barrier, preventing pathogenic bacterial antigens from entering the mucosal tissue and circulatory system. Key tight junction proteins include ZO-1 and Claudin-1. During the onset of UC, the expression levels of tight junction proteins decrease, leading to increased mucosal permeability and subsequent inflammatory responses and clinical symptoms. These were consistent with our results, indicating that the protective mechanism of Timepie001+ against UC operates by enhancing the functionality of the intestinal barrier.

UC is also considered a consequence of dysregulated immune responses due to an imbalance in the gut microbiota (Ni et al., 2017; Cheng et al., 2023; Hu et al., 2024). Patients with UC often exhibit a reduction in beneficial bacteria and an increase in harmful bacteria. Our findings consistently demonstrate that in the intestines of DSS-treated mice, there is a decrease in the diversity of specific bacterial taxa and changes in their relative abundances. Notably, in the group supplemented with Timepie001+, we observed a restoration of microbial α-diversity.

At the phylum level, studies have shown that the ratio of Firmicutes to Bacteroidetes should not be excessively high (Gomes et al., 2018). In our study, after intervention with Timepie001+, compared to the DSS group, there was an increase in Firmicutes and Bacteroidetes, and a decrease in Proteobacteria. The ratios for the NC, DSS, and Timepie001+ groups were 1.05, 1.38, and 0.52, respectively, indicating that the Timepie001+ group had a significantly lower ratio than the DSS group. At the genus level, Alistipes and Rikenella have been proved as the producer of short-chain fatty acids, which have a variety of physiological functions including provide energy for colon cells, maintain intestinal barrier integrity, regulate intestinal immunity etc., and play a significant role in intestinal homeostasis (Guo et al., 2021). The relative abundance of Alistipes and Rikenella in the intestines of mice with enteritis were severely insufficient compared to the healthy group. After taking Timepie001+, the abundance of Alistipes and Rikenella was restored to some extent. Muribaculum is a member of Muribaculaceae and has been proven to be the dominant microbiota in healthy mice in multiple studies (Lagkouvardos et al., 2016; Hiraishi et al., 2022), may participate in the prevention or alleviation of UC, but the specific mechanism is still unclear (Wang et al., 2022). The abundance of Enterorhabdus and Halomonas was significantly reduced in DSS group, which is consistent with previous research results (Tong et al., 2021; Wu et al., 2024). Meanwhile, we also noted the increase in the abundance of Pseudomonas, Bilophila, Erysipelatoclostridium and Romboutsia. Pseudomonas, a group of gram-negative bacteria, is recognized as an opportunistic pathogen associated with intestinal barrier dysfunction and infection. Overgrowth of Pseudomonas has been linked to intestinal inflammation and systemic inflammatory responses, and it is found in higher abundances in the intestines of UC patients, where it contributes to microbial imbalances and disease progression (Goldberg et al., 2008; Ren et al., 2021; Cao et al., 2024). The abundance of Bilophila, Erysipelatoclostridium, and Romboutsia have been reported to have a positive correlation with IBD (Tong et al., 2021; Cheng et al., 2023; Li et al., 2023), and our study has reached the same conclusion. Collectively, these results suggest that alterations in the gut microbiota may play a crucial role in the preventive effects of *A. muciniphila* against UC.

All results indicate that pasteurized *A. muciniphila* has a better effect on ameliorating UC than live *A. muciniphila*, which may be due to the following reasons: (1) specific active ingredients: such as Amuc_1100, the membrane proteins can interact with host cells or receptors, promote intestinal barrier function, regulate host immunity, and inhibit inflammation, thereby improving metabolic health; (2) colonization of live bacteria: the colonization of live *A. muciniphila* in the intestine may be inhibited by the host’s gut microbiota, leading to their inability to fully exert their function. Pasteurized *A. muciniphila* do not have colonization issues and can more stably exert their beneficial components. (3) Safety and stability: pasteurized *A. muciniphila* are less likely to cause infection or adverse reactions, and have higher safety.

## 5 Conclusion

This study evaluated the alleviation effects of live and pasteurized *A. muciniphila* on DSS-induced colitis in mice. The results demonstrate that pasteurized *A. muciniphila* shows a more significant improvement in UC compared to live *A. muciniphila*. Due to the fact that postbiotics do not require attention to bacterial activity and is not limited by temperature, humidity, etc., their application range is more extensive. Overall, this research provides foundational insights for the development of functional foods utilizing pasteurized *A. muciniphila* in the management of UC.

## Data Availability

The original contributions presented in the study are included in the article/supplementary material, further inquiries can be directed to the corresponding authors.

## Footnotes

^1^ http://www.ncbi.nlm.nih.gov/blast/

## References

[ref1] Astronomy and Astrophysics (1996). Guide for the care and use of laboratory animals. Washington, DC: National Academies Press. 25121211

[ref2] Berry D. Schwab C. Milinovich G. Reichert J. Ben Mahfoudh K. Decker T.. (2012). Phylotype-level 16S rRNA analysis reveals new bacterial indicators of health state in acute murine colitis. ISME J. 6, 2091–2106. doi: 10.1038/ismej.2012.39, PMID: 22572638 PMC3475367

[ref9001] Bian X. Wu W. Yang L. Lv L. Wang Q. Li Y. (2019). Administration of Akkermansia muciniphila Ameliorates Dextran Sulfate Sodium-Induced Ulcerative Colitis in Mice. Front Microbiol. 10:2259. doi: 10.3389/fmicb.2019.02259 31632373 PMC6779789

[ref3] Cao R. Fang X. Li Z. Li S. Guo Q. Chai Y. (2024). Effect of *Polygonatum sibiricum* saponins on gut microbiota of mice with ulcerative colitis. Fitoterapia 174:105855. doi: 10.1016/j.fitote.2024.105855, PMID: 38354822

[ref4] Cheng R. Xu W. Wang J. Tang Z. Zhang M. (2021). The outer membrane protein Amuc_1100 of *Akkermansia muciniphila* alleviates the depression-like behavior of depressed mice induced by chronic stress. Biochem. Biophys. Res. Commun. 566, 170–176. doi: 10.1016/j.bbrc.2021.06.018 34129964

[ref5] Cheng H. Zhang D. Wu J. Liu J. R. Tan Y. Feng W.. (2023). *Atractylodes macrocephala* Koidz. volatile oil relieves acute ulcerative colitis via regulating gut microbiota and gut microbiota metabolism. Front. Immunol. 14:1127785. doi: 10.3389/fimmu.2023.1127785, PMID: 37205093 PMC10187138

[ref6] Daglia M. Drago L. Ullah H. Di A. Brindisi G. Brunese F. P.. (2024). Effects of the supplementation of single and multi-strain probiotics, alone or in combination with other treatments, on asthma in children: a systematic review of the randomized, placebo-controlled clinical studies. J. Funct. Foods 123:106599. doi: 10.1016/j.jff.2024.106599

[ref7] Dai N. Yang X. Pan P. Zhang G. Sheng K. Wang J.. (2024). *Bacillus paralicheniformis*, an acetate-producing probiotic, alleviates ulcerative colitis via protecting the intestinal barrier and regulating the NLRP3 inflammasome. Microbiol. Res. 287:127856. doi: 10.1016/j.micres.2024.127856, PMID: 39079268

[ref8] Depommier C. Everard A. Druart C. Plovier H. Van H. M. Vieira-Silva S.. (2019). Supplementation with *Akkermansia muciniphila* in overweight and obese human volunteers: a proof-of-concept exploratory study. Nat. Med. 25, 1096–1103. doi: 10.1038/s41591-019-0495-2, PMID: 31263284 PMC6699990

[ref9] Depommier C. Hul M. V. Everard A. Delzenne N. M. Vos W. M. D. Cani P. D. (2020). Pasteurized *Akkermansia muciniphila* increases whole-body energy expenditure and fecal energy excretion in diet-induced obese mice. Gut Microbes 11, 1231–1245. doi: 10.1080/19490976.2020.1737307, PMID: 32167023 PMC7524283

[ref9002] Derrien M. Vaughan E. E. Plugge C. M. de Vos W. M. (2004). Akkermansia muciniphila gen. nov., sp. nov., a human intestinal mucin-degrading bacterium. Int J Syst Evol Microbiol. 54, 1469–1476. doi: 10.1099/ijs.0.02873-0 15388697

[ref10] Dunn K. A. Moore-Connors J. M. MacIntyre B. Stadnyk A. W. Thomas N. A. Noble A. J.. (2016). Early changes in microbial community structure are associated with sustained remission after nutritional treatment of pediatric Crohn’s disease. Inflamm. Bowel Dis. 22, 2853–2862. doi: 10.1097/MIB.0000000000000956, PMID: 27805918

[ref11] Earley H. Lennon G. Balfe I. Coffey J. C. O'Connell P. R. J. S. R. (2019). The abundance of *Akkermansia muciniphila* and its relationship with sulphated colonic mucins in health and ulcerative colitis. Sci. Rep. 9:15683. doi: 10.1038/s41598-019-51878-3, PMID: 31666581 PMC6821857

[ref12] El-Boghdady N. A. El-Hakk S. A. Abd-Elmawla M. A. (2023). The lncRNAs UCA1 and CRNDE target miR-145/TLR4/NF-қB/TNF-α axis in acetic acid-induced ulcerative colitis model: the beneficial role of 3,3-diindolylmethane. Int. Immunopharmacol. 121:110541. doi: 10.1016/j.intimp.2023.110541, PMID: 37390564

[ref13] Eslami M. Pakmehr A. Pourghazi F. Kami A. Ejtahed H.-S. Mohajeri-Tehrani M.. (2024). The anti-obesity effects of postbiotics: A systematic review of pre-clinical and clinical studies. Clin. Nutr. ESPEN 64, 370–389. doi: 10.1016/j.clnesp.2024.10.153, PMID: 39461594

[ref14] Faghfuri E. Gholizadeh P. (2024). The role of *Akkermansia muciniphila* in colorectal cancer: a double-edged sword of treatment or disease progression? Biomed. Pharmacother. 173:116416. doi: 10.1016/j.biopha.2024.116416 38471272

[ref15] Frank D. Amand A. S. S. Feldman R. Boedeker E. Harpaz N. Pace N. R. (2007). Molecular-phylogenetic characterization of microbial community imbalances in human inflammatory bowel diseases. Proc. Natl. Acad. Sci. U.S.A. 104, 13780–13785. doi: 10.1073/pnas.0706625104, PMID: 17699621 PMC1959459

[ref16] Goldberg J. B. Hancock R. E. Parales R. E. Loper J. Cornelis P. (2008). Pseudomonas 2007. J. Bacteriol. 190, 2649–2662. doi: 10.1128/jb.01950-07, PMID: 18165299 PMC2293268

[ref17] Gomes A. C. Hoffmann C. Mota J. F. (2018). The human gut microbiota: metabolism and perspective in obesity. Gut Microbes 9, 1–18. doi: 10.1080/19490976.2018.1465157, PMID: 29667480 PMC6219651

[ref18] Gómez-Gallego C. Pohl S. Salminen S. Vos W. M. D. Kneifel W. J. B. M. (2016). *Akkermansia muciniphila*: a novel functional microbe with probiotic properties. Benef. Microbe. 7, 571–584. doi: 10.3920/bm2016.0009, PMID: 27291403

[ref19] Guo C. Wang Y. Zhang S. Zhang X. Du Z. Li M.. (2021). *Crataegus pinnatifida* polysaccharide alleviates colitis via modulation of gut microbiota and SCFAs metabolism. Int. J. Biol. Macromol. 181, 357–368. doi: 10.1016/j.ijbiomac.2021.03.137, PMID: 33774071

[ref20] Han H. Ke L. Xu W. Wang H. Zhou J. Rao P. (2023). Incidental nanoparticles in black tea alleviate DSS-induced ulcerative colitis in BALB/c mice. Food Funct. 14, 8420–8430. doi: 10.1039/d3fo00641g, PMID: 37615587

[ref21] Hiraishi K. Zhao F. Kurahara L. H. Li X. Yamashita T. Hashimoto T.. (2022). Lactulose modulates the structure of gut microbiota and alleviates colitis-associated tumorigenesis. Nutrients 14:649. doi: 10.3390/nu14030649, PMID: 35277009 PMC8840163

[ref22] Hu Q. Xie J. Jiang T. Gao P. Chen Y. Zhang W.. (2024). Paeoniflorin alleviates DSS-induced ulcerative colitis by suppressing inflammation, oxidative stress, and apoptosis via regulating serum metabolites and inhibiting CDC42/JNK signaling pathway. Int. Immunopharmacol. 142:113039. doi: 10.1016/j.intimp.2024.113039, PMID: 39216118

[ref23] Jang Y. J. Kim W. K. Han D. H. Lee K. Ko G. (2019). *Lactobacillus fermentum* species ameliorate dextran sulfate sodium-induced colitis by regulating the immune response and altering gut microbiota. Gut Microbes 10, 696–711. doi: 10.1080/19490976.2019.1589281, PMID: 30939976 PMC6866707

[ref24] Lagkouvardos I. Pukall R. Abt B. Foesel B. R. U. Meier-Kolthoff J. P. Kumar N.. (2016). The mouse intestinal bacterial collection (miBC) provides host-specific insight into cultured diversity and functional potential of the gut microbiota. Nat. Microbiol. 1:16131. doi: 10.1038/nmicrobiol.2016.131, PMID: 27670113

[ref25] Lee J. Kim S. Kang C. H. (2022). Immunostimulatory activity of lactic acid bacteria cell-free supernatants through the activation of NF-κB and MAPK signaling pathways in RAW 264.7 cells. Microorganisms 10:2247. doi: 10.3390/microorganisms10112247, PMID: 36422317 PMC9698684

[ref26] Li J. Wei Y. Liu C. Guo X. Liu Z. Zhang L.. (2024). 2′-Fucosyllactose restores the intestinal mucosal barrier in ulcerative colitis by inhibiting STAT3 palmitoylation and phosphorylation. Clin. Nutr. 43, 380–394. doi: 10.1016/j.clnu.2023.12.011, PMID: 38150914

[ref27] Li K. Wu J. Xu S. Li X. Zhang Y. Gao X.-J. (2023). Rosmarinic acid alleviates intestinal inflammatory damage and inhibits endoplasmic reticulum stress and smooth muscle contraction abnormalities in intestinal tissues by regulating gut microbiota. Microbiol. Spectr. 11:e0191423. doi: 10.1128/spectrum.01914-23, PMID: 37594285 PMC10654191

[ref28] Li Y. Yue X. Ren X. Pang Y. Wang T. Huangfu B.. (2024). Mare milk and fermented mare milk alleviate DSS-induced ulcerative colitis in mice by reducing inflammation and modulating intestinal flora. J. Dairy Sci. doi: 10.3168/jds.2024-25181, PMID: 39647629

[ref29] Ma S. Yeom J. Lim Y. H. (2020). Dairy *Propionibacterium freudenreichii* ameliorates acute colitis by stimulating MUC2 expression in intestinal goblet cell in a DSS-induced colitis rat model. Sci. Rep. 10:5523. doi: 10.1038/s41598-020-62497-8, PMID: 32218552 PMC7099060

[ref30] Madsen K. L. Malfair D. Gray D. Doyle J. S. Jewell L. D. Fedorak R. N. J. I. B. D. (2010). Interleukin-10 gene-deficient mice develop a primary intestinal permeability defect in response to enteric microflora. Inflamm. Bowel Dis. 5, 262–270. doi: 10.1002/ibd.3780050405 10579119

[ref31] Miao Y. Wang M. Sun H. Zhang Y. Zhou W. Yang W.. (2024). *Akkermansia muciniphila* ameliorates colonic injury in mice with DSS-induced acute colitis by blocking macrophage pro-inflammatory phenotype switching via the HDAC5/DAB2 axis. Biochim. Biophys. Acta 1871:119751. doi: 10.1016/j.bbamcr.2024.119751, PMID: 38776988

[ref32] Neurath M. F. (2014). Cytokines in inflammatory bowel disease. Nat. Rev. Immunol. 14, 329–342. doi: 10.1038/nri3661, PMID: 24751956

[ref33] Ni J. Wu G. D. Albenberg L. Tomov V. T. (2017). Gut microbiota and IBD: causation or correlation? Nat. Rev. Gastroenterol. Hepatol. 14, 573–584. doi: 10.1038/nrgastro.2017.88, PMID: 28743984 PMC5880536

[ref34] Ouwerkerk J. P. Aalvink S. Belzer C. de Vos W. M. (2016). *Akkermansia glycaniphila* sp. nov., an anaerobic mucin-degrading bacterium isolated from reticulated python faeces. Int. J. Syst. Evol. Microbiol. 66, 4614–4620. doi: 10.1099/ijsem.0.001399, PMID: 27499019

[ref35] Payahoo L. Khajebishak Y. Alivand M. R. Soleimanzade H. Alipour S. Barzegari A.. (2019). Investigation the effect of oleoylethanolamide supplementation on the abundance of *Akkermansia muciniphila* bacterium and the dietary intakes in people with obesity: a randomized clinical trial. Appetite 141:104301. doi: 10.1016/j.appet.2019.05.032, PMID: 31132422

[ref9003] Qu S. Fan L. Qi Y. Xu C. Hu Y. Chen S. (2021). Akkermansia muciniphila Alleviates Dextran Sulfate Sodium (DSS)-Induced Acute Colitis by NLRP3 Activation. Microbiol Spectr. 9:e0073021. doi: 10.1128/Spectrum.00730-21 34612661 PMC8510245

[ref36] Ren Z. Pan L. L. Huang Y. Chen H. Liu Y. Liu H.. (2021). Gut microbiota-CRAMP axis shapes intestinal barrier function and immune responses in dietary gluten-induced enteropathy. EMBO Mol. Med. 13:e14059. doi: 10.15252/emmm.202114059, PMID: 34125490 PMC8350901

[ref37] Seni K. Saini A. Debnath R. Singh A. Sharma A. Bisht D. S.. (2024). Advancements in ulcerative colitis management: a critical assessment of etrasimod therapy. Health Sci. Rev. 12:100196. doi: 10.1016/j.hsr.2024.100196

[ref38] Seregin S. S. Golovchenko N. Schaf B. Chen J. Pudlo N. A. Mitchell J.. (2017). NLRP6 protects IL10^−/−^ mice from colitis by limiting colonization of *Akkermansia muciniphila*. Cell Rep. 19, 733–745. doi: 10.1016/j.celrep.2017.03.080, PMID: 28445725 PMC5528001

[ref39] Sharon G. Ioannis K. Willem D. V. Clara B. J. M. (2018). *Akkermansia muciniphila* in the human gastrointestinal tract: when, where, and how? Microorganisms 6, 75–81. doi: 10.3390/microorganisms6030075, PMID: 30041463 PMC6163243

[ref40] Shen Z. H. Zhu C. X. Quan Y. S. Yang Z. Y. Wu S. Luo W. W.. (2018). Relationship between intestinal microbiota and ulcerative colitis: mechanisms and clinical application of probiotics and fecal microbiota transplantation. Gastroenterology 24, 5–14. doi: 10.3748/wjg.v24.i1.5, PMID: 29358877 PMC5757125

[ref41] Shi D.-C. Wang P.-Y. Xu L. Zhu H. Zhang W.-Y. Wu Q.-Y.. (2024). Potential of *Dendrobium officinale* oligosaccharides to alleviate chronic colitis by modulating inflammation and gut microbiota. Food Med. Homol. 2024:9420077. doi: 10.26599/fmh.2025.9420077

[ref42] Tong L. Hao H. Zhang Z. Lv Y. Yi H. J. T. (2021). Milk-derived extracellular vesicles alleviate ulcerative colitis by regulating the gut immunity and reshaping the gut microbiota. Theranostics 11, 8570–8586. doi: 10.7150/thno.62046, PMID: 34373759 PMC8344018

[ref43] Turuvekere Sadguruprasad L. Basavaraj M. (2018). Statistical modelling for optimized lyophilization of *Lactobacillus acidophilus* strains for improved viability and stability using response surface methodology. AMB Express 8:129. doi: 10.1186/s13568-018-0659-3, PMID: 30097787 PMC6086920

[ref44] Vigsnaes L. Brynskov J. Steenholdt C. Wilcks A. Licht T. (2012). Gram-negative bacteria account for main differences between faecal microbiota from patients with ulcerative colitis and healthy controls. Benefic. Microbes 3, 287–297. doi: 10.3920/BM2012.0018, PMID: 22968374

[ref45] Wang H. Cai Y. Wu W. Zhang M. Dai Y. Wang Q. (2024). Exploring the role of gut microbiome in autoimmune diseases: a comprehensive review. Autoimmun. Rev. 23:103654. doi: 10.1016/j.autrev.2024.103654, PMID: 39384149

[ref46] Wang Y. Chen Y. Zhang H. Yu S. Yuan G. Hu H. (2025). Colon-targeted self-assembled nanoparticles loaded with berberine double salt ameliorate ulcerative colitis by improving intestinal mucosal barrier and gut microbiota. Colloids Surf. B 245:114353. doi: 10.1016/j.colsurfb.2024.114353, PMID: 39509850

[ref47] Wang J. L. Han X. Li J. X. Shi R. Liu L. L. Wang K.. (2022). Differential analysis of intestinal microbiota and metabolites in mice with dextran sulfate sodium-induced colitis. World J. Gastroenterol. 28, 6109–6130. doi: 10.3748/wjg.v28.i43.6109, PMID: 36483152 PMC9724481

[ref48] Wang J. Lv X. Li Y. Wu H. Chen M. Yu H.. (2025). A ROS-responsive hydrogel that targets inflamed mucosa to relieve ulcerative colitis by reversing intestinal mucosal barrier loss. J. Control. Release 377, 606–618. doi: 10.1016/j.jconrel.2024.11.065, PMID: 39608456

[ref49] Wang L. Tang L. Feng Y. Zhao S. Han M. Zhang C.. (2020). A purified membrane protein from *Akkermansia muciniphila* or the pasteurised bacterium blunts colitis associated tumourigenesis by modulation of CD8^+^ T cells in mice. Gut 69, 1988–1997. doi: 10.1136/gutjnl-2019-320105, PMID: 32169907 PMC7569398

[ref50] Wang D. Xu R. Liu S. Sun X. Zhang T. Shi L.. (2025). Enhancing the application of probiotics in probiotic food products from the perspective of improving stress resistance by regulating cell physiological function: A review. Food Res. Int. 199:115369. doi: 10.1016/j.foodres.2024.115369, PMID: 39658167

[ref51] Wangchuk P. Yeshi K. Loukas A. (2024). Ulcerative colitis: clinical biomarkers, therapeutic targets, and emerging treatments. Trends Pharmacol. Sci. 45, 892–903. doi: 10.1016/j.tips.2024.08.003, PMID: 39261229

[ref52] Wegh C. A. M. Geerlings S. Y. Knol J. Roeselers G. Belzer C. (2019). Postbiotics and their potential applications in early life nutrition and beyond. Int. J. Mol. Sci. 20:4673. doi: 10.3390/ijms20194673, PMID: 31547172 PMC6801921

[ref53] Wu Y. Jha R. Li A. Liu H. Zhang Z. Zhang C.. (2022). Probiotics (*Lactobacillus plantarum* HNU082) supplementation relieves ulcerative colitis by affecting intestinal barrier functions, immunity-related gene expression, gut microbiota, and metabolic pathways in mice. Microbiol. Spectr. 10:e0165122. doi: 10.1128/spectrum.01651-22, PMID: 36321893 PMC9769980

[ref54] Wu Z. Li Y. Jiang M. Sang L. Chang B. (2024). Selenium yeast alleviates dextran sulfate sodium-induced chronic colitis in mice by reducing proinflammatory cytokines and regulating the gut microbiota and their metabolites. J. Inflamm. Res. 17, 2023–2037. doi: 10.2147/JIR.S449335, PMID: 38577691 PMC10992675

[ref55] Xiao X. Wu Y. Jie Z. Lin L. Li Y. Hu W.. (2024). *Akkermansia Muciniphila* supplementation improves hyperlipidemia, cardiac function, and gut microbiota in high fat fed apolipoprotein E-deficient mice. Prostaglandins Other Lipid Mediat. 175:106906. doi: 10.1016/j.prostaglandins.2024.106906, PMID: 39265779

[ref56] Yao B. Yang Z. Zhao X. Han Z. Li P. Shang N. (2025). Biofilm-state probiotics: advanced alternatives to traditional probiotics. Trends Food Sci. Technol. 156:104854. doi: 10.1016/j.tifs.2024.104854

[ref57] Yu E. Eid J. Cheng A. Lynch B. Bauter M. (2024). Lack of genotoxicity and subchronic toxicity in safety assessment studies of *Akkermansia muciniphila* formulation. Toxicol. Rep. 13:101790. doi: 10.1016/j.toxrep.2024.101790, PMID: 39554606 PMC11565037

[ref58] Zhai R. Xue X. Zhang L. Yang X. Zhao L. Zhang C. (2019). Strain-specific anti-inflammatory properties of two *Akkermansia muciniphila* strains on chronic colitis in mice. Front. Cell. Infect. Microbiol. 9:239. doi: 10.3389/fcimb.2019.00239, PMID: 31334133 PMC6624636

[ref59] Zhang S.-s. Feng D. An J.-z. Zhao J. Zhao J.-y. Guo Y.. (2025). Total glycosides of *Cistanche deserticola* attenuates DSS-induced inflammatory bowel disease by regulating intestinal environmental homeostasis. Food Med. Homol. 2:9420048. doi: 10.26599/fmh.2025.9420048

[ref60] Zhang Q. Wu Y. Wang J. Wu G. Long W. Xue Z.. (2016). Accelerated dysbiosis of gut microbiota during aggravation of DSS-induced colitis by a butyrate-producing bacterium. Sci. Rep. 6:27572. doi: 10.1038/srep27572, PMID: 27264309 PMC4893749

[ref61] Zhang K. Zhang Q. Qiu H. Ma Y. Hou N. Zhang J.. (2024). The complex link between the gut microbiome and obesity-associated metabolic disorders: mechanisms and therapeutic opportunities. Heliyon 10:e37609. doi: 10.1016/j.heliyon.2024.e37609, PMID: 39290267 PMC11407058

[ref62] Zhao X. Zhao J. Li D. Yang H. Chen C. Qin M.. (2023). *Akkermansia muciniphila*: a potential target and pending issues for oncotherapy. Pharmacol. Res. 196:106916. doi: 10.1016/j.phrs.2023.106916, PMID: 37690533

[ref63] Zheng M. Han R. Yuan Y. Xing Y. Zhang W. Sun Z.. (2023). The role of *Akkermansia muciniphila* in inflammatory bowel disease: current knowledge and perspectives. Front. Immunol. 13. doi: 10.3389/fimmu.2022.1089600, PMID: 36685588 PMC9853388

[ref64] Zhou J.-C. Zhang X.-W. (2019). *Akkermansia muciniphila*: a promising target for the therapy of metabolic syndrome and related diseases. Chin. J. Nat. Med. 17, 835–841. doi: 10.1016/S1875-5364(19)30101-3, PMID: 31831130

